# Characterization of the blastogenic response to LPS of bovine peripheral blood mononuclear cells

**DOI:** 10.1371/journal.pone.0204827

**Published:** 2018-10-02

**Authors:** Massimo Amadori, Joel Fernando Soares-Filipe, Federica Riva, Andrea Vitali, Jessica Ruggeri, Nicola Lacetera

**Affiliations:** 1 Laboratory of Cellular Immunology, Istituto Zooprofilattico Sperimentale della Lombardia e dell’Emilia-Romagna (IZSLER), Brescia, Italy; 2 Department of Veterinary Medicine (DIMEVET), UNIMI, Milan, Italy; 3 University of Tuscia, Department of Agriculture and Forestry Science (DAFNE), Viterbo, Italy; University of Illinois, UNITED STATES

## Abstract

Mitogens are diverse compounds of plant and microbial origin, widely employed to test immunocompetence in animals. The blastogenic response of bovine Peripheral Blood Mononuclear Cells (PBMC) to lypopolysaccharides **(**LPS) has been investigated in our laboratories for a long time. In particular, a possible correlation between blastogenic response to LPS and disease resistance of periparturient dairy cows had been observed in previous studies. Most important, low responder cows presented a higher frequency of disease cases after calving, compared with high responder animals. Owing to the above, different aspects of the blastogenic response to LPS were investigated on PBMC of healthy Friesian cows, using a 72-hour Bromodeoxyuridin (BrDU) cell proliferation assay. Stimulation with LPS induced little if any replication of bovine PBMC over 72 hours despite consistent BrDU detection in all the PBMC samples under study. Poor replication of LPS-stimulated PBMC was confirmed by cell cycle and cell growth flow cytometry analyses. In particular, LPS stimulation gave rise to very low percentages of S phase cells, sometimes lower than in control, unstimulated cells, as opposed to Concanavalin A-stimulated PBMC. Magnetic separation and analysis of BrDU-treated bovine PBMC after exposure to LPS showed that both B and CD4 T cells are involved in the blastogenic response to LPS, in contrast with current data based on human and murine models. Finally, LPS caused an early, specific up-regulation of TNF-α and TLR4 genes in bovine PBMC, and significant correlations were shown between the expression of inflammatory cytokine and Indoleamine-pyrrole 2,3-dioxygenase (IDO1) genes. On the whole, our data indicate that differences in the blastogenic response to LPS could be partly accounted for by heterogenicity of responding cells (B and T lymphocytes), which might also have an impact on induction and regulation of inflammatory responses and endotoxin tolerance.

## Introduction

Mitogens are diverse compounds of plant and microbial origin, widely employed to test immunocompetence in animals. In healthy, non-immunocompromised hosts, they induce DNA synthesis and division of large leucocyte populations, which can be reasonably associated with immunologic competence of T or B cells. Accordingly, mitogens are usually employed in diverse lymphocyte proliferation tests. Among these, liquid scintillation counting after ^3^H-thymidine incorporation has been the reference assay over many years, but the stepwise reduction of radioisotope usage has prompted the development and refinement of alternative assays like ELISAs for Bromodeoxyuridine (BrDU), flow-cytometry-based procedures based on Carboxyfluorescein succinimidyl ester (CFSE), DNA-intercalating fluorochromes like propidium iodide, Ki-67 nuclear antigen, as well as 3-(4,5-dimethylthiazol-2-yl)-2,5-diphenyltetrazolium bromide (MTT)-based and cell counting procedures (see [1], for review).

Mitogens are frequently classified in terms of mitogen-reactive leukocyte population. On this basis, mitogens are classified as T cell specific, B cell specific or polyspecific. T cell mitogens, alone or in combination, include Phorbol 12-myristate 13-acetate (PMA), ionomycin, A23187, Phytohemagglutinin (PHA), Concanavalin A (Con A), anti-CD3 Ab, anti-TcR αβ Ab, anti-TcR γδ Ab, Staphylococcal toxins A, B and E. B cell mitogens include anti-IgM Ab, lipopolysaccharides (LPS), 8-mercaptoguanosine, protein kinase C activators, calcium ionophores, dextran sulfate, polyinosinic:polycytidylic acid (PolyIC), to name a few. Instead, Pokeweed Mitogen (PWM) can induce proliferation of both T and B cells [1].

The blastogenic response of bovine Peripheral Blood Mononuclear Cells (PBMC) to LPS has been investigated for a long time in our laboratories because of fundamental points of interest. In particular, a possible correlation between blastogenic response to LPS and disease resistance of periparturient dairy cows was surmised due to the findings generated by our previous study [2]. First, there is strong evidence of a physiologically-regulated responsiveness of PBMC to LPS, with a significant, stepwise decrease of the response in dairy cows after calving. Secondly, dairy cows can be classified as high, medium and low responders, and the relative distance between these levels remains pretty much the same in the framework of the above down-regulation of the response after calving. Most important, low responder cows presented particular haplotypes of the TLR4 gene and a higher frequency of disease cases after calving, compared with high responder animals [2]. Albeit the limited number of cows in this latter study demands an independent confirmation of our test results, yet the possible implications of these on fundamental features of animal health and welfare make a case for further characterization of this assay in the target species, cattle.

Owing to the above, we set out to perform a characterization of the proliferative response to LPS in cattle, aiming to define its fundamental features and the responding lymphocyte populations.

## Materials and methods

### Samplings

Twenty-five Holstein Friesian, healthy dairy cows of two experimental farms were enrolled in this study. The health status was evaluated on the basis of clinical inspection and somatic cell counts (SCC) in milk. This study complied with Italian laws on animal experimentation and ethics. The procedures involving animals and their care abode with protocols approved by the Animal Care Units of Institute of Zootechnics, Piacenza, and Università degli Studi di Milano and by the Italian Ministry of Health (authorizations 1047/2015-PR and 628/2016-PR) in compliance with national (D.L. n.26, March 4, 2014) and European policies (EEC Council Directive 2010/63/UE). 5-ml blood samples were obtained from the jugular vein and all efforts were made to minimize the number of animals used and their suffering. No further types of sampling and/or additional animal handling were applied to cows. Blood samples were transported at room temperature and processed within six hours after collection. Days in milk (DIM) ranged in one farm between 288 and 320 (average = 301.4, SD = 11.9); parity ranged between 1 and 4 (average = 2.4, SD = 0.9). In the other farm DIM ranged between 104 and 274 (average = 146.9, SD 68); parity ranged between 1 and 4 (average = 2.2, SD = 0.7). The above time points were beyond the usual boundaries of the negative energy balance (NEB) after calving.

Animals were housed in a loose housing system during the dry period and, after parturition, in a tie-stall housing system. Cows were milked twice daily and fed *ad libitum* with a total mixed ration without silage, using alfalfa hay, straw and concentrated feed with mineral and vitamin supplementations.

### Bovine PBMC

PBMC were isolated from venous blood by density gradient centrifugation as described previously [3]. Briefly, venous blood diluted in PBS was layered over Ficoll-Paque PLUS medium (APB, Milan, Italy) and centrifuged at 600 × *g*, 18°C for 45 min. The mononuclear cell band was recovered and washed twice in PBS. Residual red blood cells were eliminated by hypotonic shock using redistilled water. Cells were resuspended at 2x10^6^ viable cells/mL in RPMI 1640 medium, Hepes 20 mM, with 2 mM glutamine, supplemented with 10%, heat-inactivated fetal calf serum (FCS), 100 U penicillin, 100 μg streptomycin, and 0.25 μg/mL amphotericin B (Sigma). Cell viability was assessed by a Trypan Blue exclusion test carried out either in a 0.1 mm Bürker chamber, or in Countess Automated Cell Counter (ThermoFisher Scientific), according to the manufacturer’s directions.

### In vitro proliferation of bovine PBMC

Briefly, 0.1-mL aliquots of PBMC (see above) were reacted in triplicate in 96-well microtiter plates with 0.1 ml LPS (from *Escherichia coli* O111:B4; Sigma, 40 μg/mL), or with medium only (control), and incubated at 39°C in 5% CO_2_. The concentration of PBMC was therefore 10^6^ / ml final. After 48 hours, 20 μl/well of 100 μM BrdU in culture medium were added to the wells, and cell proliferation was quantified after an additional 18-hour incubation, using the Cell Proliferation ELISA BrDU (colorimetric) kit (Roche, cat. 11647229001), according to the manufacturer’s directions. Triplicate control wells contained the PBMC suspension without LPS or BrdU. Values of PBMC proliferation were expressed as the mean optical density (OD) for test wells minus the OD for control wells without BrdU. The LPS concentration adopted in the proliferation assay (20 μg/mL final) had been shown to cause the highest release of TNF-α in bovine monocytes [4]. On the basis of this report, we tested LPS concentrations of 0.1, 1, 10 e 20 μg/ml in bovine PBMC, and no significant differences were observed in terms of proliferation (Lacetera N., unpublished results). Therefore, the highest LPS concentration (20 μg/mL final) was deemed convenient for both proliferation assays and investigations into bovine inflammatory cytokine responses. The above BrDU assays were carried out on 16 cows.

PBMC were also grown at the same concentration (10^6^ / ml final) in 25 cm^2^ plastic bottles with or without LPS (20 μg/mL final). In this case, PBMC proliferation after 3 to 6 days was evaluated by CFSE staining [1], or by staining nuclear antigen Ki-67 with fluorescein isothiocyanate (FITC)-Mouse anti-Ki-67 Set (BD Pharmingen, cat. 556026), according to the manufacturer’s directions, using a Guava EasyCyte HT flow cytometer with Incyte software. Ki-67 is a nuclear protein, strictly associated with cell proliferation. The monoclonal antibody to human Ki-67 antigen had been previously characterized as cross-reactive with its bovine orthologue (Amadori M., unpublished results). The CFSE and Ki-67 detection assays were carried out on 8 cows.

### Cell cycle assay

The relationship between data of BrDU proliferation assays and DNA duplication under the above-mentioned test conditions was investigated on aliquots of fresh bovine PBMC. The BrDU assay was carried out on PBMC grown in 96-well plates over 72 hours. At the same time, non-adherent PBMC (lymphocytes) in 6-well plates were collected from LPS-stimulated and control wells. Bovine PBMC were also grown with Con A (2.5 μg/mL final) under the same conditions of LPS-stimulated cells for comparative purposes. After extensive washings in PBS, cells were fixed overnight in 70% ethanol at -20°C, and then stained with propidium iodide (PI) in the presence of RNAse I, as previously described [5]. Cells were analyzed using software “Cell Cycle” in a Guava EasyCyte HT flow cytometer. This procedure was used to discriminate between resting G1, G2/M and S-phase cells in LPS-stimulated and control PBMC.

The cell cycle assays were carried out on 4 cows.

### Cell growth assay

The cell growth assay was carried out on 2 cows. Bovine PBMC were stained with CFSE as previously described [1] and grown with or without LPS (20 μg/mL final) in 25 cm^2^ plastic bottles over 3 to 6 days at 39°C. Next, cells were washed with PBS and resuspended in 0.5 mM EDTA/PBS for a further incubation of 5 min at 37°C. Then, cells were pelleted, resuspended in 0.2 mL of flow cytometry buffer (PBS with 2% heat-inactivated FCS and 0.1% azide) and reacted with 10 microliters of PI (50 μg/mL). After a 5-min incubation at 4°C, cells were analyzed in a Guava EasyCyte HT flow cytometer using the Cell Growth procedure, which discriminates CFSE and PI-stained cells. The gating dot plot of forward scatter versus green fluorescence allowed to select both resting and proliferating cells, and to discard debris from the analysis. The analysis dot plot included green (CFSE) versus red fluorescence (PI) with proper quadrant markers. Live proliferating cells (CFSE low, PI-) were thus discriminated from live non-proliferating cells (CFSE high, PI-) and non-viable, non-proliferating cells (CFSE high, PI+).

### Freezing of LPS and BrDU-stimulated bovine PBMC

PBMC of eight healthy cows were separated and stimulated as described above with LPS, followed by addition of BrDU in 75-cm^2^ plastic tissue culture bottles over 72 hours. Control cultures without LPS were set up as well. Non-adherent cells were centrifuged (330 g, 10’, 20°C), washed in medium without serum and frozen at -80°C in aliquots of 4x10^6^ cells in RPMI 1640 medium supplemented with 40% FCS and 10% dimethyl sulfoxide (DMSO). Cells were employed over the following 6 months for separation and immunophenotyping purposes.

### Magnetic separation and analysis of BrDU-treated and frozen bovine PBMC

Three frozen PBMC aliquots (4x10^6^ each) of eight cows were thawed at 38°C and washed twice in degassed PBS + 0.5% bovine serum albumin (BSA) + 2 mM EDTA (separation buffer). Two PBMC aliquots had been stimulated with LPS, as opposed to the third one (control). Each aliquot was pelleted and resuspended in 0.1 mL of separation buffer. LPS-stimulated cells were separately reacted with one or more of the following monoclonal antibodies: IL-A30, anti-bovine sIgM (B cell-specific) [6]; IL-A12, anti-bovine CD4 [7]; IL-A51, anti-bovine CD8 [8], obtained as a generous gift of Dr. Wilma Ponti (University of Milan, Italy); MM1A, anti-CD3 (pan-T) [9], purchased from VMRD (Pullman, Washington, USA). Unstimulated control cells were reacted with both IL-A30 and one of the T cell specific mAb. After 30 min at 4°C, cells were pelleted and resuspended with 35 microliters of separation buffer (final volume: 80 μL). 20 μL of MACS anti-mouse IgG microbeads (Miltenyi Biotec, cat. 130-048-402) were added to LPS-stimulated aliquots, only. These were incubated for a further 15 minutes at 4°C. 0.1 mL of goat anti-mouse IgG1-Phycoerythrin (PE) (for mAb IL-A30, MM1A and IL-A51) or goat anti-mouse IgG2a-PE (for mAb IL-A12) were added to LPS-stimulated and control cells. Goat anti-mouse IgG (H+L) Alexa Fluor 488 replaced both PE conjugates in a couple of experiments. Cells were incubated for another 5’ at 4°C. Cells were washed once and resuspended in 0.5 mL of separation buffer. Then, each aliquot was separated on MACS MS columns (Miltenyi Biotec 130-042-201) according to the manufacturer’s directions. Positively and negatively selected cells, as well as non-separated, control cells, were analyzed in a Guava EasyCyte HT flow cytometer using Incyte software. This enabled us to evaluate the efficiency of separation and to measure the cell concentration in each sample. Then, 3x10^5^ viable cells/well of positively and negatively selected cells in 0.1 mL separation buffer were seeded into 96-well tissue culture plates, dried at 60°C and analyzed by the Cell Proliferation ELISA BrDU (colorimetric) kit.

### Gene expression studies

PBMC of other eight healthy, lactating dairy cows were grown in 6-well tissue culture plates (4x10^6^ cells/well, 10^6^/mL) and stimulated with LPS (1 or 20 μg/mL), or left as untreated controls over 4 or 20 hours. Next, non-adherent cells were collected, pelleted (330 *g*, 10’, 5°C), lysed in 1.0 mL TRI Reagent (Sigma-Aldrich, St. Louis, MO, USA)/well together with the plastic-adherent ones, and frozen at -80°C; total RNA was then extracted according to the manufacturer’s directions. Total RNA was extracted from uncultured PBMC, as well. The concentration and quality of RNA was determined using a spectrophotometer (BioPhotometer, Eppendorf, Hamburg, Germany) at 260/280 nm wavelength. Total RNA (1 μg) was reverse-transcribed using the High Capacity cDNA Reverse Transcription Kit (Applied Biosystem, Foster City, CA, USA), according to the manufacturer’s instructions. The cDNA obtained from each sample was used as a template for Real-Time PCR in an optimized 25 μL reaction volume using Sybr Green chemicals, as previously described [10]. Samples were then used to measure the expression of some genes involved in the inflammatory response by Real-Time quantitative PCR: TLR4 (Toll-like receptor 4), Interleukin(IL)1-β, IL-6, Tumor Necrosis Factor (TNF)-α, Indoleamine-pyrrole 2,3-dioxygenase (IDO)1, tryptophan 2,3-dioxygenase (TDO)2. Glyceraldehyde-3-phosphate dehydrogenase (GAPDH) was investigated as housekeeping gene. Primer pair sequences are listed in Table 1. The primers were purchased from Invitrogen (Carlsbad, CA, USA). Real-Time quantitative PCR was carried out in the 7000 Sequence Detection System (Applied Biosystems, Foster City, CA, USA), as previously described [10]. Each sample was amplified by Real-Time PCR in duplicate. The expression of bovine target genes was normalized using the calculated GAPDH cDNA expression (mean) of the same sample and run. The relative quantification of each gene was calculated by the “Delta Ct” method [11]. The obtained value was multiplied by 10,000 in order to obtain the test Arbitrary Units.

**Table 1:** Oligonucleotide primer sequences for SYBR Green quantitative RT-polymerase chain reaction amplification.

*Gene*	*Protein*	*Sequence*	*Gene Bank* *accession number*	*Efficiency*	*Slope*	*Ct range*
IL-1β	Interleukin-1 beta	F: CTGTTATTTGAGGCTGATGACC R: TTGTTGTAGAACTGGTGAGAAATC	GI:27806570	99.4%	-3.32	16.8–21.6
TNF-α	Tumor Necrosis Factor alpha	F: TCTTCTCAAGCCTCAAGTAACAAGT R: CCA TGA GGG CAT TGG CAT AC	GI:402693442	99.2%	-3.34	21.6–25.1
IL-6	Interleukin-6	F: CAC TCCAGAGAAAACCGAAGC R: GAAGCATCCCGTCCTTTTCCTC	GI:161579163	101%	-3.29	20.4–28.1
TLR4	Toll-like receptor 4	F: CTGCGGCTCTGATCCCAG R: TTAGGAACAACCTGTACGCAAGG	GI: 19744156	106.7%	-3.17	27.8–31.5
IDO1	Indoleamine 2,3-dioxygenase	F: GGGTCAAGGCGATGGAGAC R: ACAGCGATATTGCTTGGCAA	GI:402744521	98.8%	-3.35	19.8–27.6
TDO2	Tryptophan 2,3-dioxygenase	F: TTGAGGCATGGCTGGAAAG R: AGTTAAATCCATGCGGCTCTAAAC	GI:114052634	103.5%	-3.24	25.6–35.2
GAPDH	Glyceraldehyde-3-phosphate dehydrogenase	F: GGCGTGAACCACGAGAAGTATAA R: CCCTCCACGATGCCAAAGT	GI:89573946	94.4%	-3.46	19.5–25

F = forward primer; R = reverse primer. Efficiency = -1+10(-1/slope).

Slopes between -3.1 and -3.6, giving reaction efficiencies between 90 and 110% are typically acceptable.

### Statistical analyses

One-way ANOVA and Newman-Koels post test were applied to the data sets of BrDU, cell cycle and cell growth assays. Significant prevalence differences between lymphocyte populations in flow cytometry analyses were checked by Fisher’s exact test on 10,000 events. A Student’s *t* test was carried out on mean values of uncultured, cultured unstimulated and cultured, LPS-stimulated PBMC, respectively, after pairwise comparisons of data from RT Real-Time PCR assays. The significance threshold was set at P< 0.05. Finally, statistical tests were used to reveal bivarial linear correlations between IDO1 or TDO2 gene expression and cytokines (IL-1β, TNF-α, IL-6) and TLR4 expression, as well as correlations among cytokines (IL-1β, TNF-α, IL-6) at 4 hours of stimulation with 1 μg/mL LPS. Since the levels of expression of all the genes under study did not show a normal distribution (Shapiro-Wilk test), correlation was computed with the non-parametric two-tails rho test of Spearman with α = 0.05.

## Results

### Stimulation with LPS induces little if any replication of bovine PBMC

Nine healthy, lactating dairy cows were submitted to the LPS-stimulation assay in microtiter plates and tissue culture flasks performed immediately after separation of PBMC (Table 2). Whereas all the animals tested positive for BrDU, only 3 out of nine cattle showed a slight increase (≥ 10%) of both cell counts and % vitality after a 72-hour culture with respect to the values before LPS stimulation.

**Table 2:** Reactivity of bovine PBMC to LPS stimulation.

Increased cell count*	No increase of cell count	Decreased cell count**
3 out of 9 cows	4 out of 9 cows	2 out of 9 cows

Venous blood was collected from nine healthy lactating dairy cows. PBMC were immediately separated and submitted to a 72-hour LPS stimulation assay. Results were evaluated in terms of viable cell counts with a Trypan Blau exclusion test. BrDU incorporation was checked in five out of nine cows. All the five cows were BrDU-positive.

* ≥ 10% viable cells with respect to unstimulated control.

**≤ 10% with respect to unstimulated control.

### Poor replication of LPS-stimulated PBMC is confirmed by cell cycle, cell growth and Ki-67 analyses

**Fig 1:**
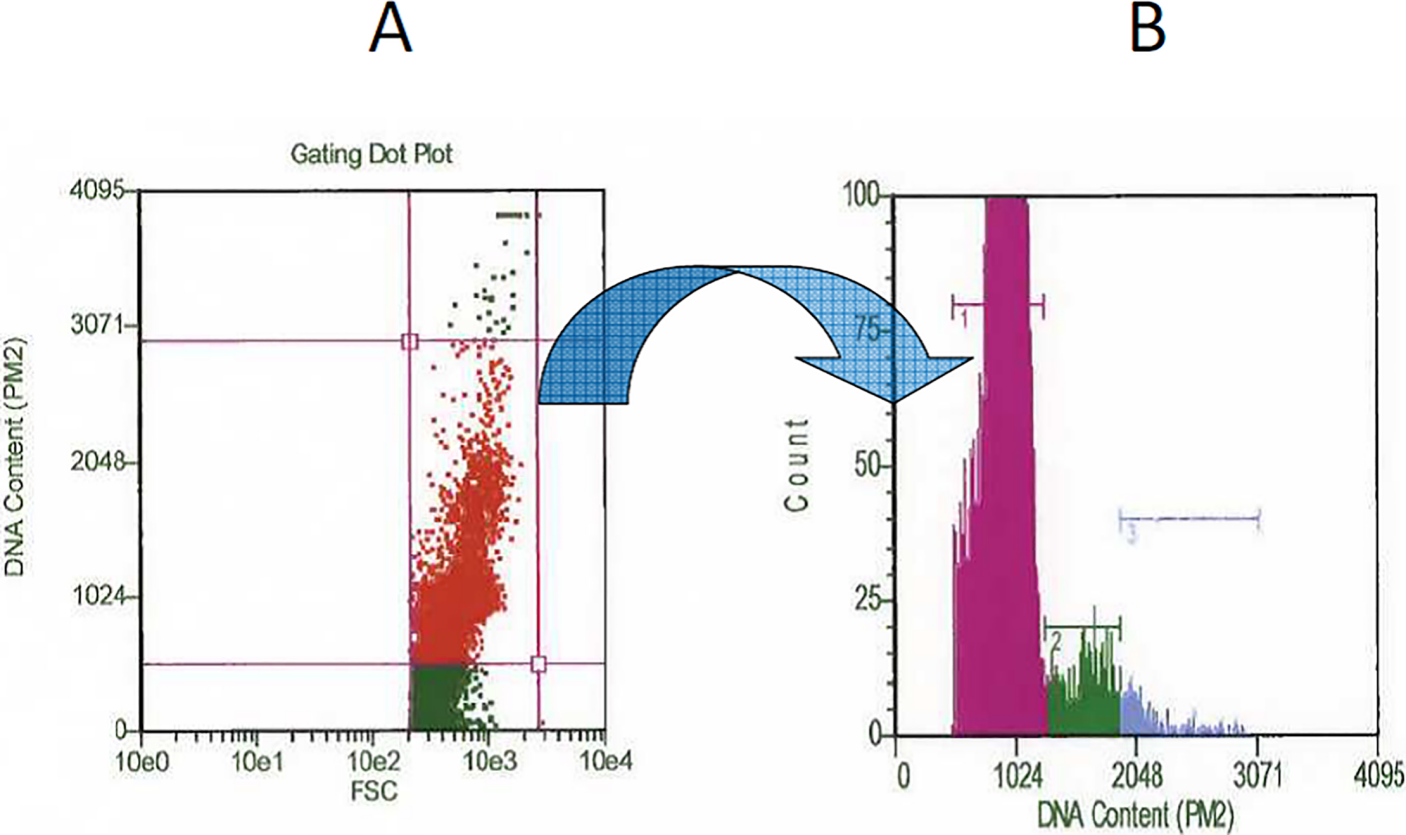
Cell cycle assay. PBMC were separated from venous blood of two dairy cows and immediately submitted to a LPS stimulation assay in 25 cm^2^-flasks. After three days in culture, non-adherent PBMC (lymphocytes) were fixed overnight at -20°C in 70% ethanol and stained with PI in the presence of RNAse for 30 min at room temperature. PBMC were analyzed in a Guava EasyCyte HT flow cytometer using software “Cell Cycle” (Merck Millipore). Panel A: cells were analyzed by a combination of forward scatter (FSC) and PI incorporation. Red-colored cells were thus gated to the analysis histogram. Panel B: analysis histogram. Region 1, G1 phase cells; region 2, S phase cells; region 3, G2/M phase cells. The results obtained on one cow are shown, and these were similar to those of the other animal (see S1 Table).

**Fig 2:**
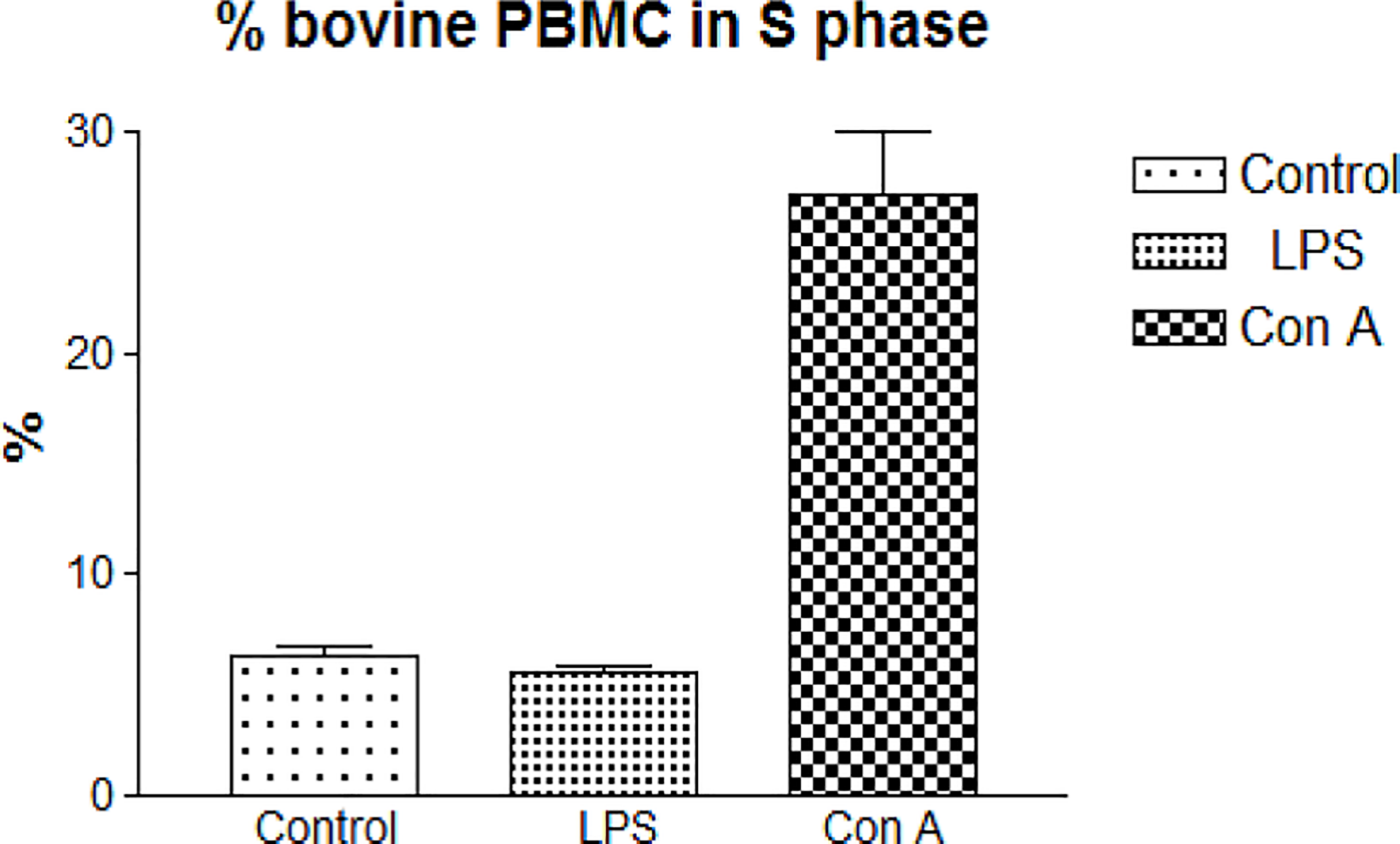
LPS and Con A-stimulated bovine PBMC. PBMC were separated from venous blood of one cow and immediately submitted to LPS and Con A stimulation assays, respectively. After three days in culture, non-adherent PBMC (lymphocytes) were fixed overnight at -20°C in 70% ethanol and stained with PI in the presence of RNAse for 30 min at room temperature. PBMC were analyzed in a Guava EasyCyte HT flow cytometer using software “Cell Cycle” (Merck Millipore). The percentages of S phase cells of control, LPS-stimulated and Con A-stimulated PBMC are shown as mean ± 1 standard deviation of three test replicates. The percentage of S phase cells was significantly higher after Con A stimulation (P< 0.001). There was no significant difference instead between control and LPS-stimulated PBMC.

The poor responses in terms of cell counts prompted us to investigate the cell cycle of control and LPS-stimulated bovine PBMC. LPS stimulation gave rise to very low percentages of S and G2/M phase cells (Fig 1 and S1 Table), as opposed to Con A-stimulated PBMC (Fig 2). In particular, the two animals in Fig 1 did not show increased cell counts after LPS treatment (see Table 2).

**Fig 3:**
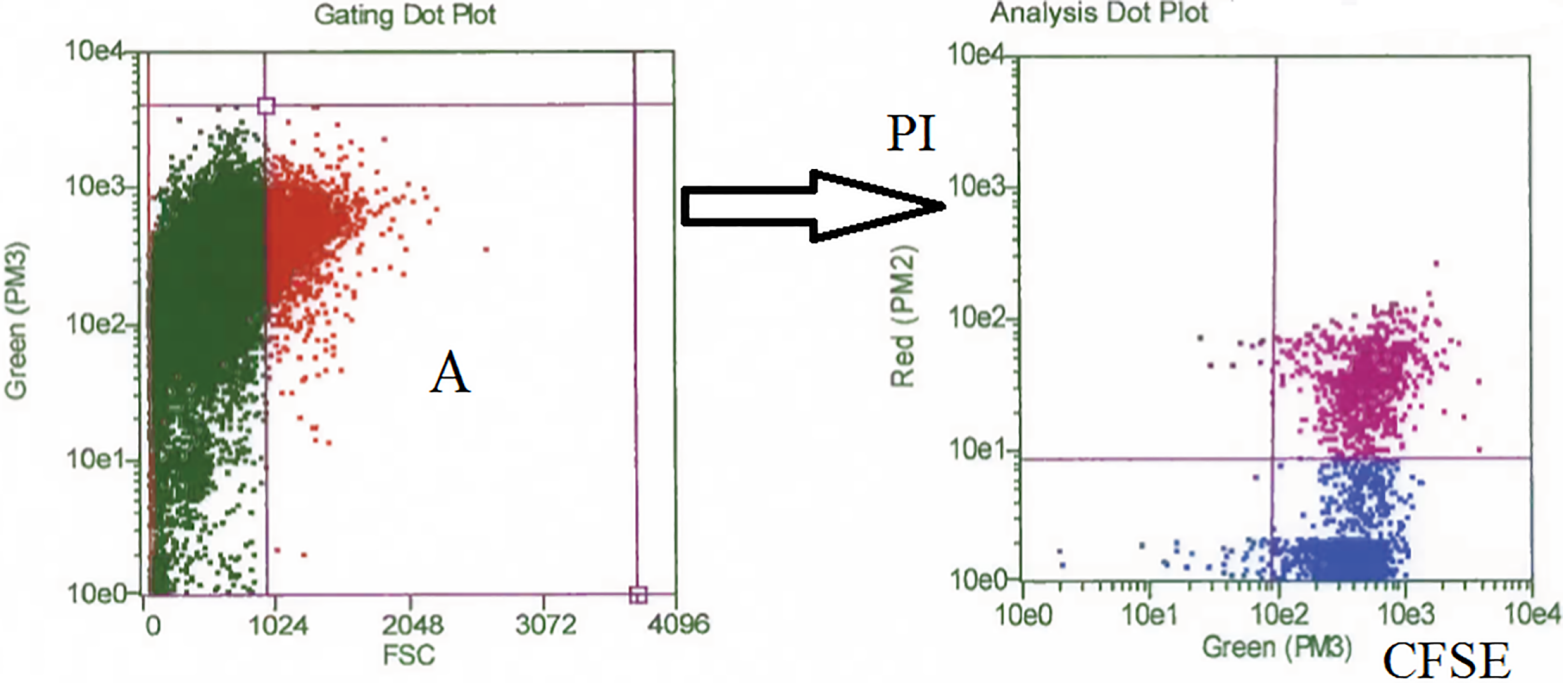
Cell growth assay. PBMC were separated from venous blood of two dairy cows, stained with CFSE and grown in medium supplemented with LPS (20 μg/mL final) or kept as unstimulated control in 25 cm^2^ plastic bottles over 3 days at 39°C. After treatment with 0.5 mM EDTA in PBS, non-adherent PBMC (lymphocytes) were pelleted, resuspended in 0.2 mL of flow cytometry buffer and reacted with 10 μL of PI (50 micrograms/mL). After a 5-min incubation at 4°C, cells were analyzed in a Guava EasyCyte HT flow cytometry using the Cell Growth software, which discriminates CFSE and PI-stained cells. The gating dot plot of forward scatter versus green fluorescence allowed to select both resting and proliferating cells, and to discard debris from the analysis (left, panel A). Red-colored cells (panel A) were gated to the analysis dot plot (right). This included green (CFSE) versus red fluorescence (PI) with proper quadrant markers. Live proliferating cells (CFSE low, PI-negative) of a representative experiment are shown in the lower left quadrant. Similar results were obtained on lymphocytes of the other cow under study.

These results were substantially in agreement with cell counts in microtiter plates (Table 2), and were confirmed by a cell growth study on two cows (Fig 3 and S2 Table), which demonstrated low percentages of live, replicating PBMC after LPS stimulation (4.32 and 1.75% in cow 1 and 2, respectively), similar to those of control, unstimulated PBMC. Most cells were instead live, but not proliferating (67.86 and 54.65%, respectively), at the end of the 3-day in vitro culture with LPS. Finally, cultured PBMC of 2 other cows were analyzed for expression of nuclear Ki-67 antigen after 48 hours. This was expressed by 2.9 and 1.7% of LPS-stimulated PBMC, as opposed to 1.5 and 1% of control cells (data not shown). On the whole, all our results pointed at a low prevalence of replicating cells following LPS stimulation despite consistent BrDU detection in PBMC cultures.

### Both B and CD4 T cells are involved in the response of bovine PBMC to LPS

**Fig 4:**
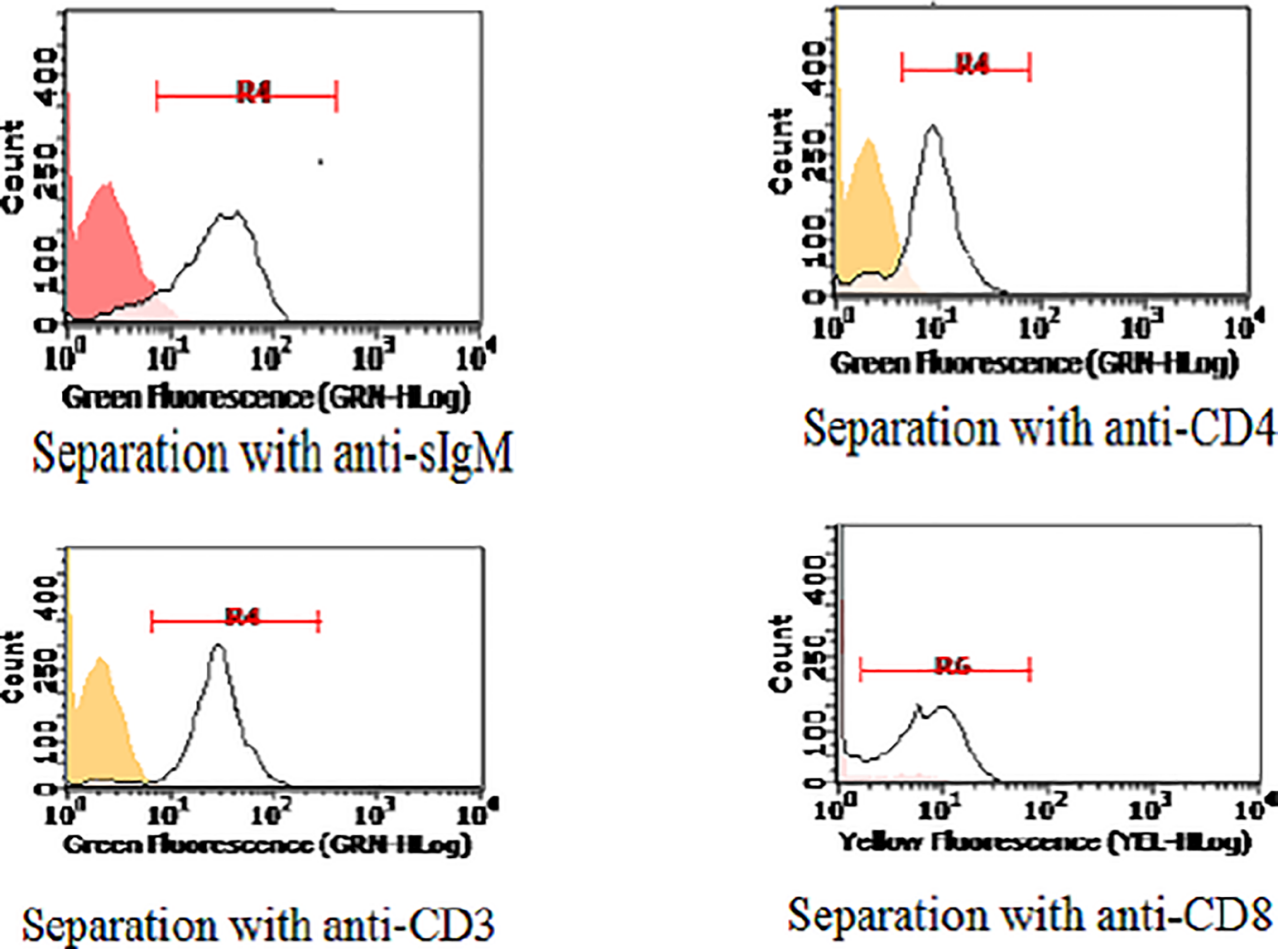
Magnetic separation with monoclonal antibodies. LPS-stimulated and BrDU-treated, as well as untreated PBMC of eight cows were frozen at -80°C. Next, they were thawed and separately reacted with one of the following monoclonal antibodies: IL-A30 (anti-bovine sIgM, B cell-specific), IL-A12 (anti-bovine CD4), IL-A51 (anti-bovine CD8), MM1A (anti-bovine CD3, pan-T). After 30 min at 4°C, cells were washed and 20 microliters of MACS anti-mouse IgG microbeads were added. After 15 minutes at 4°C, cells were reacted for a further 5 minutes with either PE or Alexa Fluor 488 anti-mouse IgG conjugates. Next, cells were submitted to magnetic separation on MACS MS columns according to the manufacturer’s directions. Cells were washed once, resuspended in 0.5 mL MACS separation buffer and analyzed in a Guava EasyCyte HT flow cytometer using Incyte software. Representative results of positive selections are shown. Unseparated cells stained with anti-IgG conjugates only served as negative control. On the whole, the assays were carried out on PBMC of eight cows and 16 samples, i.e. BrDU-treated and untreated PBMC of each cow.

The above results also showed moderate differences of BrDU signal among PBMC samples. Accordingly, we wondered if heterogenicity of LPS-responding leukocytes could partly account for the observed levels of incorporation. Therefore, PBMC of eight healthy dairy cattle were stimulated with LPS in tissue culture flasks or kept as untreated control, and then frozen in aliquots at -80°C after BrDU treatment. Cells were thawed at 38°C and submitted to magnetic separation with different monoclonal antibodies (Fig 4). First, PBMC of three cattle were separated with mAb to surface bovine IgM (sIgM, B cells) and CD8 (CD8 T cells). Results showed a consistent BrDU signal in B cells and CD8-negative PBMC. Next, 2 other PBMC samples were separated with mAb to surface bovine IgM (B cells) and CD4 (CD4 T cells). The two samples were BrDU-positive in both negative and positive fractions of sIgM and CD4-separated PBMC. These results were confirmed on two other PBMC samples. Finally, the same experiment was repeated on the eighth, last PBMC sample, using mAb to sIgM and CD3 (pan T) for magnetic separation, to confirm that bovine T cells are involved in the proliferative response to LPS. Results clearly indicated substantial BrDU incorporation into both negative and positive fractions of sIgM and CD3-separated PBMC. Since frozen PBMC samples contained a rather high percentage of dead cells (20 to 40%) which might affect the efficiency of separation, the above results were checked on fresh PBMC samples of three other healthy, lactating dairy cattle. Both CD3-positive and negative PBMC showed a BrDU signal (cow 1) and the same was demonstrated after separation with anti-sIgM and CD4 mAb (cows 2 and 3) (see Table 3 for a summary of all the results).

**Table 3:** BrDU ELISA on bovine PBMC fractions obtained by magnetic cell separation.

	BrDU incorporation in PBMC fractions obtained by magnetic cell sorting			
	CD3 (pan T)	CD4 (T cells)	CD8 (T cells)	sIgM (B cells)
**Magnetic (+) fraction**	+	+	-	+
**Non-magnetic (-)** **fraction**	+	+	+	+

LPS-stimulated and BrDU-treated bovine PBMC were submitted to magnetic cell sorting with mAb to CD3, CD4, CD8 (T cells) and surface IgM (sIgM, B cells). Magnetic (mAb-positive) and non-magnetic (mAb-negative) fractions were analyzed for BrDU with an ELISA kit. Results were obtained on BrDU-treated PBMC of 9 cows.

A comparative analysis of BrDU incorporation was carried out on PBMC of 6 cows, other than those shown in Table 2; three cows had higher BrDU incorporation into B cells, two of them into CD4 T cells, whereas one presented comparable levels of incorporation in both cell populations (data not shown).

### Bovine B and CD4+ T cells may proliferate after stimulation with LPS

**Fig 5:**
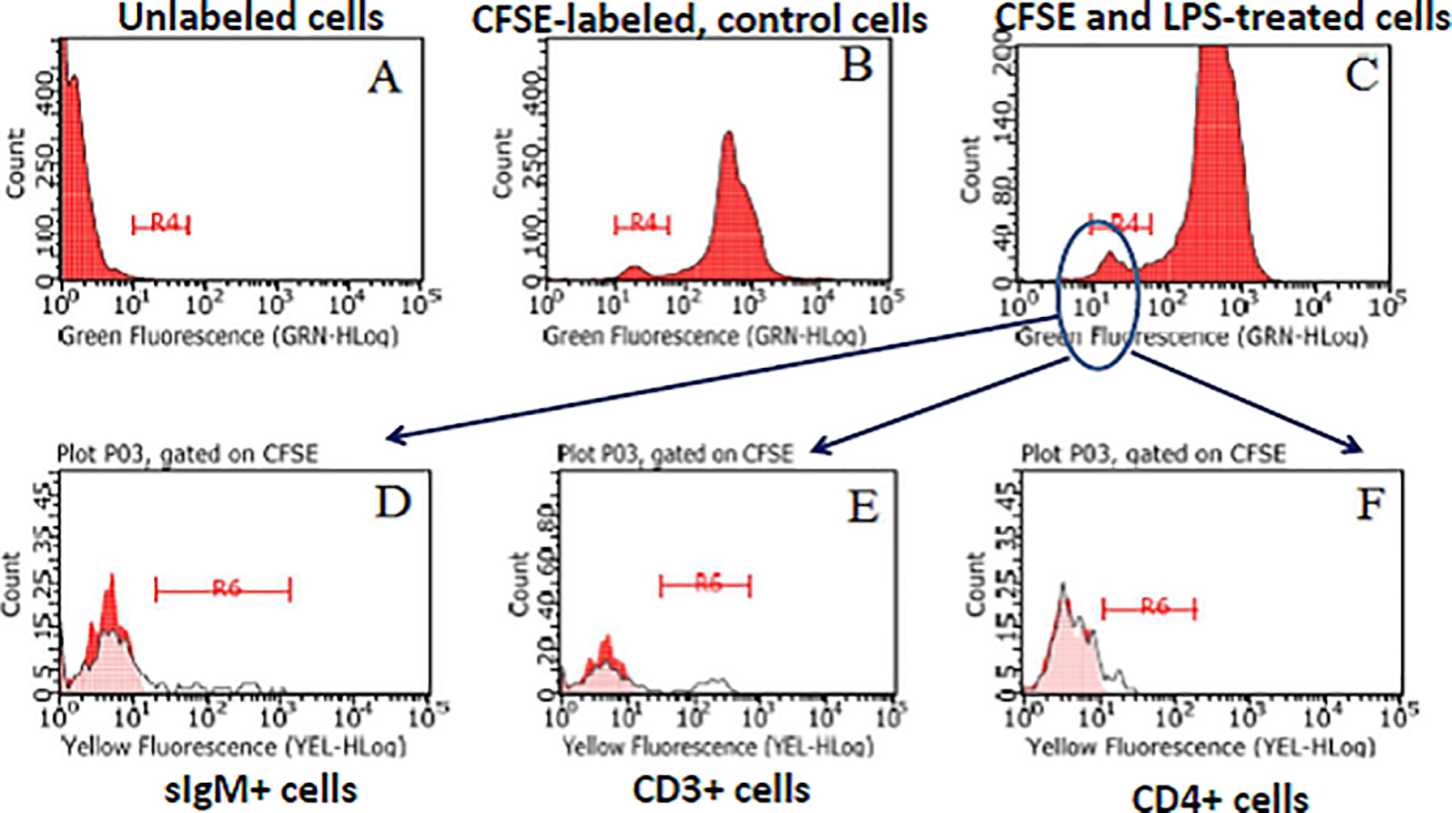
CFSE staining of bovine PBMC. Bovine PBMC from 6 cows were separated from heparinized blood, immediately labelled with CFSE and either stimulated with LPS or kept as untreated control in three distinct experiments. One no CFSE / no LPS control was set up as well. After 3 or six days in culture, non-adherent PBMC (lymphocytes) were pelleted and analyzed. In three experiments on 4 cows, lymphocytes were stained with mAb to bovine CD3, CD4 and sIgM, followed by anti-mouse IgG1 PE or anti-mouse IgG2 PE. Proliferated cells (halved green fluorescence) defined a gate (region R4) for the evaluation of CD3, CD4 and IgM surface expression against the background (cells reacted with either anti-mouse IgG1 PE or anti-mouse IgG2 PE, only). The results of one representative experiment are shown. Panels. A: unlabeled cells. B: CFSE-labeled, control cells. C: CFSE-labeled, LPS-stimulated cells. After gating in region R4 (proliferating, CFSE-low cells) of LPS-stimulated cells (panel C), expression of sIgM, CD3 and CD4 is shown in panels D, E and F, respectively (see also S3 Table).

Our results had shown that a response to LPS in terms of BrDU incorporation could be mounted by both B and CD4 T cells. This prompted us to investigate possible differences between the two cell populations in terms of cell division (CFSE staining) of fresh PBMC. Representative results of one experiment out of three are shown in Fig 5. In agreement with our previous results, the prevalence of CFSE-positive cells was not significantly different between control and LPS-stimulated cells. As expected, CFSE-low, LPS-stimulated PBMC included both B and T cells (Fig 5). Please notice however that bovine CD4 undergoes a stepwise down-regulation in cultured PBMC, which can affect the flow cytometry estimate.

### Correlation between expression of kynurenine pathway and inflammatory cytokine genes after stimulation with LPS

**Fig 6:**
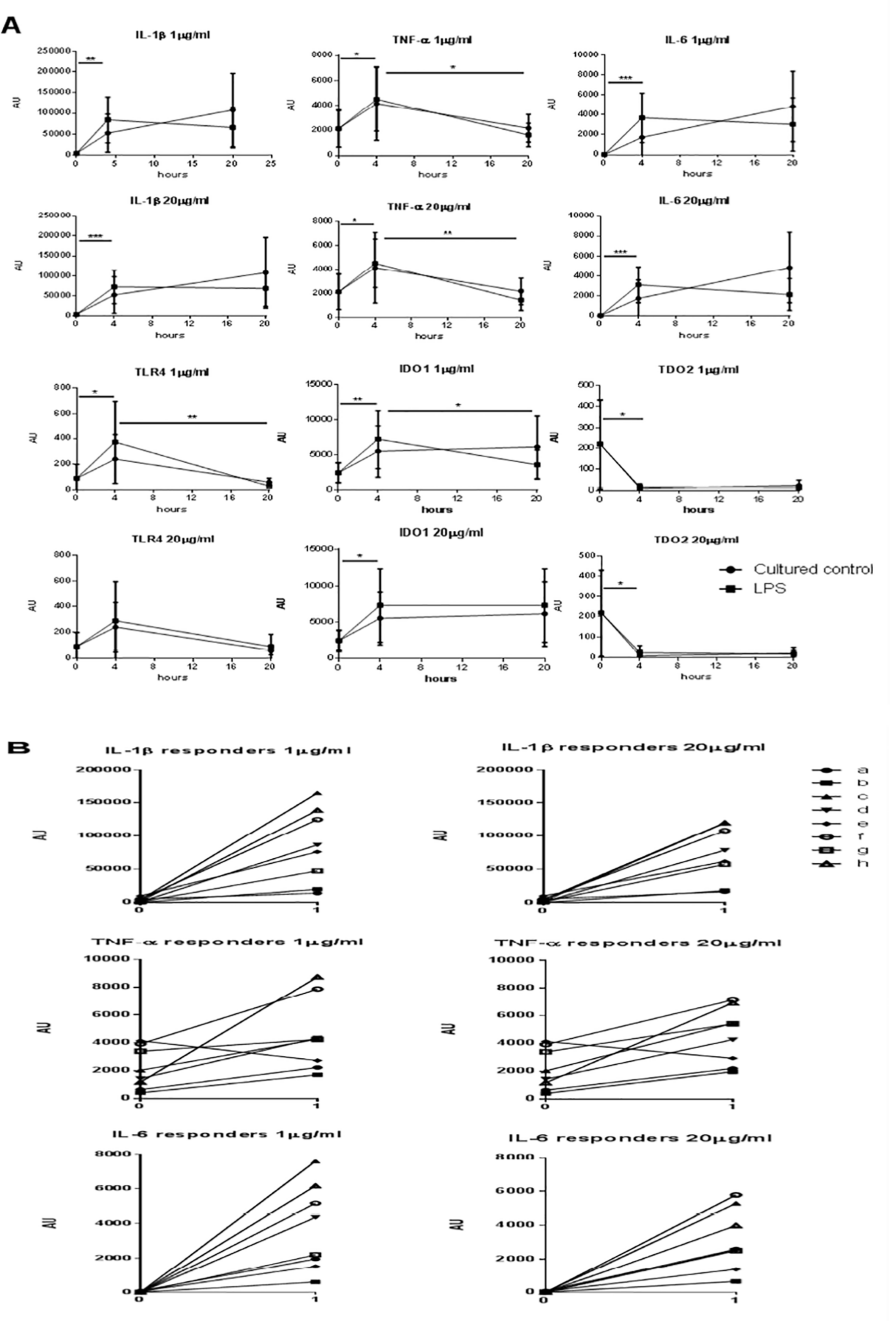
Expression of inflammatory cytokine and kynurenine pathway genes. IL-1β, TNF-α, IL-6, TLR4, IDO1 and TDO2 mRNA expression was analyzed by Real Time PCR in PBMC of 8 healthy cows. **Panel A.** PBMC were separated and immediately cultured; they were analyzed at 0 hour (uncultured control), at 4 and 20 hours with three concentrations of LPS (0, 1 and 20 μg/mL). The gene expression level of all genes was normalized to GAPDH and the results are presented as Arbitrary Units. A Student’s *t* test was applied between the groups: 0 vs 4 hours; control vs LPS-stimulated at each time point; 4 hours vs 20 hours. The reported horizontal bars and asterisks refer to LPS-stimulated cultures, only (* = p< 0.05; ** = p< 0.005; *** = p< 0.0005). **Panel B.** Expression levels of IL-1β, TNF-α, IL-6 of PBMC from all tested animals (named: a, b, c, d, e, f, g, h), uncultured (time point 0) and at 4 hours of stimulation with 1 μg/ml of LPS. On the whole, our results refer to 8 cows and 40 samples (8 samples at 0 hour, uncultured; 16 samples with 1 μg/ml LPS at 4 and 20 hours, respectively; 16 samples with 20 μg/ml LPS at 4 and 20 hours, respectively).

In order to get an insight into the inflammatory response to LPS we decided to investigate the expression of the main inflammatory cytokine genes and kynurenine pathway genes involved in endotoxin tolerance [12] in other eight healthy cows (Fig 6, panels A and B), not used in the previous tests.

In particular, we compared gene expression at the LPS concentration of our assay vs. a much lower one (1 μg/mL), used in other studies on the *in vitro* inflammatory response. As shown in Fig 6 (panel A), a significant up-regulation of all the genes under study was observed at 4 hours in LPS-stimulated cultures compared to uncultured cells from the same animal. The same was observed in control PBMC cultures with the exception of TNF-α and TLR4 genes, which were significantly up-regulated by the LPS treatment, only. On the whole, no significant differences were observed at each time point between LPS-stimulated and control cultures, and neither between cultures stimulated with 1 and 20 μg/mL LPS, respectively. These only differed in the time-course of IDO1 expression, decreasing at 20 hours with 1 but not with 20 μg/mL LPS (Fig 6). As for uncultured PBMC, wide differences were observed in the expression of the TDO2 gene, varying from nought to very high levels (data not shown). As expected, there was a significant positive correlation between the expressions of inflammatory cytokine genes in LPS-stimulated cultures (1 μg/mL) at 4 hours: IL-1β vs. IL-6 p = 0.0072 / IL-1β vs TNF-α p = 0.0218 / IL-6 vs TNF-α p = 0.0154. Moreover, in LPS-stimulated cultures (1 μg/mL), IDO1 was significantly correlated with IL-6 gene expression at 20 hours (p = 0.0458). As shown in Fig 6 (panel B), the eight cows under study could be nicely discriminated between low, medium and high responders in terms of IL-1β and IL-6 expression at 4 hours in LPS-stimulated cultures.

## Discussion

Although mitogens have been largely employed in veterinary immunology, most data about their target cells have been obtained in human and murine models. These provided contradictory data about the target cells of LPS in the proliferation assays. An absolute specificity of this assay for B cells of mice has been surmised for a long time [13], but not confirmed in humans: human, but not murine memory CD8+ T cells can play in fact an important role in the response to LPS by expressing both TLR4 and CD14 receptors [14]. In addition to that, a complex interplay exists between proliferative response to LPS of lymphocytes and inflammatory response of monocytes/macrophages. In particular, the response to LPS of B cells is regulated by RP105 (CD180). This receptor is functionally associated with TLR4, and B cells of RP105-KO mice show a reduced response to LPS. On the contrary, monocytes of the same KO mice show stronger inflammatory responses to LPS [15]. Thus, RP105 is involved in positive and negative regulation of LPS–driven responses in B cells and macrophages, respectively.

Three main features had been highlighted in our previous study on dairy cows [2]: (A) high, medium and low responders in the LPS proliferation assay could be consistently detected; (B) there was a stepwise, overall reduction of the proliferative response after calving, but the differences between the three patterns of response were substantially unaffected; (C) high and low responder showed lower and higher prevalence of disease cases, respectively, after calving, which was seemingly associated with particular haplotypes of the TLR4 gene. If our data should be confirmed on a greater number of animals, a potent, robust effector mechanism of innate immunity is likely to be associated with the reactivity of cattle in the LPS assay *in vitro*. Accordingly, we were prompted to investigate the possible reasons underlying the different profiles of response. In this respect, a crucial issue to be dealt with was the actual lymphocyte population of cattle involved in the *in vitro* blastogenic response to LPS. If the responding cells were B lymphocytes, the negative regulation of the blastogenic response could be accounted for by the increased levels of Non-Esterified Fatty Acids (NEFA) after calving [16], in accordance with an established murine model [17].

Owing to the above, we decided to investigate the possible responder cells in cattle on the basis of the previous data in murine and human models. Whereas the response of B cells was clearly confirmed by our data, we could substantially rule out a possible role of CD8+ T cells and highlight instead an important co-response of CD4+ T cells, which is therefore peculiar to bovine PBMC to the best of our knowledge. Also, our data showed a different possible involvement of both lymphocyte populations in the response to LPS in the cows under study, which may partly account for the different levels of response in the BrDU assay. This might be related to a different prevalence of LPS-reactive CD4 T cells, expressing both TLR4 and CD14 receptors, like human CD8+ memory T cells [14]. The role of such a response and the balance between responses to LPS of B cells, T cells and macrophages in cattle should be defined in the future in a suitable *in vitro* model.

Despite consistent responses in the BrDU assay, the actual numerical increase of responding cells was always low or undetectable, as opposed to stimulation with Con A. In particular, there was a very good agreement between cell counts, cell cycle and cell growth assays, and expression of nuclear Ki-67 antigen. This implies a discrepancy between nucleotide incorporation and downstream cell cycle block. This is probably related to the double signaling pathway activated by TLR4, i.e. the MYD88/NFκB and TRIF/IRF3 pathway, respectively [18]. The latter can lead to sustained induction of Type I (mainly α/β interferons, which may exert potent anti-proliferative effector functions [19]. We do not rule out other possible mechanisms. In particular, IDO1 is involved in activation of the amino acid-sensitive general control, non-depressible 2 (GCN2) stress kinase pathway; this can elevate IL-6 and CC chemokine ligand 2 (CCL2) and lead to cell cycle arrest [20]. The correlation observed in our study between IL-6 and IDO1 gene expression is in agreement with this latter mechanism.

It should be stressed that our protocol made use of a high concentration of LPS (20 μg/mL) to investigate the proliferative responses, on the basis of the aforementioned validation. With regard to the test conditions in our assay, a high dose as 20 micrograms LPS per ml allowed for a correct discrimination between different levels of response, although the same peaks of proliferation can be reached (as in other species) using much lower LPS concentrations (see Materials and Methods section). Therefore, we decided to investigate possible genetic markers of such a high-dose LPS treatment. With respect to control conditions, only TNF-α and TLR4 genes were significantly up-regulated by LPS in cultured PBMC. This was not surprising because of the possible non-specific effects on *in vitro* expression of inflammatory cytokine genes [21,22]. Also, the higher LPS concentration (20 μg/mL) differed from the lower one (1 μg/mL) only in terms of time-course of IDO1 expression (Fig 6).

Interestingly, the response of bovine PBMC to LPS in terms of inflammatory cytokine gene expression (Fig 6) was clearly reminiscent of the high/medium/low responding profiles in terms of blastogenic response observed in our previous study. Among inflammatory cytokine genes, IL-6 expression was clearly correlated with that of IDO1, i.e. with the kynurenine and aryl hydrocarbon receptor(AhR)-based endotoxin tolerance pathway [12]. In this respect, the high levels of TDO2 gene expression observed in uncultured PBMC of some healthy cows could imply an important role of this pathway for the maintenance of homeostasis after exposure to environmental stressors.

Owing to the above, the higher disease prevalence of LPS low-responder cows [2] might be the outcome of two distinct, but possibly overlapping phenomena. On the one hand, a reduced blastogenic response to LPS could be somehow correlated with abnormal profiles of endotoxin tolerance, observed in diverse disease cases [23]. On the other hand, low proliferative responses of lymphocytes to LPS might correspond to increased inflammatory responses in monocytes/macrophages, possibly related to low RP105 expression [15]. Vice versa, high proliferative responses of PBMC due to high RP105 expression could be associated with a more effective control of the inflammatory responses in the pregnancy to lactation transition period, which might underlie improved clinical scores of dairy cows.

## Conclusions

The above hypotheses should be checked in future studies, in which the proliferative response to LPS of PBMC from lactating dairy cows at different DIM should be investigated in association with the actual levels of RP105 expression, inflammatory cytokine gene expression, inflammometabolic parameters *in vivo* [16], and the *in vitro* inflammatory response to products of metabolic stress like NEFA and β-hydroxybutyrate (BHB). Most important, future studies should investigate the expression of IL-1beta and IL-6 genes and proteins in samples from cows used in LPS/BrDU experiments to see if clusters of cows with greater inflammatory responses were the same or not as those identified by BrDU labeling.

In this scenario, the levels of blastogenic response to LPS could prove to be a useful prognostic parameter of the host’s coping ability vis-à-vis environmental, infectious and non-infectious stressors [24].

## Supporting information

S1 Table Cell cycle and BrDU proliferation assays. PBMC were separated from venous blood of two dairy cows and immediately submitted to a LPS and BrDU stimulation assay in 96-well microtiter plates. PBMC were also stimulated with LPS only in 25 cm^2^ flasks. After three days in culture, non-adherent PBMC (lymphocytes) of the flasks were fixed overnight at -20°C in 70% ethanol, stained with PI and analyzed in a Guava EasyCyte HT flow cytometer using software “Cell Cycle” (Merck Millipore).
(DOCX)

S2 Table Cell growth assay. PBMC were separated from venous blood of two dairy cows, stained with CFSE and grown in medium supplemented with LPS (20 μg/mL final) or kept as unstimulated control in 25 cm^2^ plastic bottles over 3 days at 39°C. Cells were analyzed in a Guava EasyCyte HT flow cytometry using the Cell Growth software (Merck Millipore), which discriminates live, dead, proliferating, non-proliferating cells after staining with PI.
(DOCX)

S3 Table Staining of bovine PBMC after CFSE labeling and LPS stimulation. In three experiments on 4 cows, PBMC were immediately labelled with CFSE and either stimulated with LPS or kept as untreated control. After 3 to 6 days in culture, lymphocytes were stained with mAb to bovine CD3, CD4 and sIgM, followed by anti-mouse IgG1 PE or anti-mouse IgG2 PE.
(DOCX)
