# Supplementary material for: Characterization of the blastogenic response to LPS of bovine peripheral blood mononuclear cells
Source: PLoS One. 2018 Oct 2;13(10):e0204827. doi: 10.1371/journal.pone.0204827 (PMC6168128; doi:10.1371/journal.pone.0204827)
Supplement: S1 Table — PBMC were separated from venous blood of two dairy cows and immediately submitted to a LPS and BrDU stimulation assay in 96-well microtiter plates. PBMC were also stimulated with LPS only in 25 cm2 flasks. After three days in culture, non-adherent PBMC (lymphocytes) of the flasks were fixed overnight at -20°C in 70% ethanol, stained with PI and analyzed in a Guava EasyCyte HT flow cytometer using software “Cell Cycle” (Merck Millipore). (DOCX) [file pone.0204827.s001.docx]

**S Table 1**

**Cell cycle and BrDU proliferation assays**

1. **BrDU assay**

|  | **OD control**  **(no LPS)** | **OD stimulated** | **OD**  **no BrDU control** | **OD**  **medium** |
| --- | --- | --- | --- | --- |
| **Cow 1** | 0.121±0.008 | 0.212±0.005 | 0.097±0.034 | 0.095±0.021 |
| **Cow 2** | 0.118±0.022 | 0.229±0.015 | 0.078±0.005 | 0.099±0.029 |

Results are shown in terms of OD 450nm of 3 test replicates ± 1 SD

1. **Cell cycle assay**

|  | **Cow 1**  **% G1 cells** | **Cow 1**  **% G2-M cells** | **Cow 2**  **% G1 cells** | **Cow 2**  **% G2-M cells** |
| --- | --- | --- | --- | --- |
| **Control, no LPS** | **87.35** | **7.20** | **84.63** | **3.79** |
| **LPS-stimulated** | **88.71** | **6.69** | **76.75** | **4.95** |

PBMC were separated from venous blood of two dairy cows and immediately submitted to a LPS and BrDU stimulation assay in 96-well microtiter plates. PBMC were also stimulated with LPS only in 25 cm^2^ flasks. After three days in culture, non-adherent PBMC (lymphocytes) of the flasks were fixed overnight at -20°C in 70% ethanol and stained with PI in the presence of RNAse for 30 min at room temperature. PBMC were analyzed in a Guava EasyCyte HT flow cytometer using software “Cell Cycle” (Merck Millipore).
