# Supplementary material for: Characterization of the blastogenic response to LPS of bovine peripheral blood mononuclear cells
Source: PLoS One. 2018 Oct 2;13(10):e0204827. doi: 10.1371/journal.pone.0204827 (PMC6168128; doi:10.1371/journal.pone.0204827)
Supplement: S2 Table — PBMC were separated from venous blood of two dairy cows, stained with CFSE and grown in medium supplemented with LPS (20 μg/mL final) or kept as unstimulated control in 25 cm2 plastic bottles over 3 days at 39°C. Cells were analyzed in a Guava EasyCyte HT flow cytometry using the Cell Growth software (Merck Millipore), which discriminates live, dead, proliferating, non-proliferating cells after staining with PI. (DOCX) [file pone.0204827.s002.docx]

**S Table 2**

**Cell growth assay**

|  | **% CFSE-, PI-** | **% CFSE-, PI+** | **% CFSE+, PI-** | **% CFSE+, PI+** |
| --- | --- | --- | --- | --- |
| **Cow 1**  **control** | 68.05 | 28.85 | 2.65 | 0.45 |
| **Cow 1**  **LPS-treated** | 67.86 | 27.41 | 4.32 | 0.40 |
| **Cow 2**  **control** | 58.25 | 36.0 | 5 | 0.75 |
| **Cow 2**  **LPS-treated** | 54.65 | 42.65 | 1.75 | 0.95 |

PBMC were separated from venous blood of two dairy cows, stained with CFSE and grown in medium supplemented with LPS (20 μg/mL final) or kept as unstimulated control in 25 cm^2^ plastic bottles over 3 days at 39°C. After treatment with 0.5 mM EDTA in PBS, non-adherent PBMC (lymphocytes) were pelleted, resuspended in 0.2 mL of flow cytometry buffer and reacted with 10 μL of PI (50 micrograms/mL). After a 5-min incubation at 4°C, cells were analyzed in a Guava EasyCyte HT flow cytometry using the Cell Growth software, which discriminates CFSE and PI-stained cells.

CFSE+: cells with halved green fluorescence after mitosis. CFSE-: cells with full green fluorescence.

PI+: dead cells. PI-: live cells. CFSE+, PI- : live proliferating PBMC.
