# Supplementary material for: Characterization of the blastogenic response to LPS of bovine peripheral blood mononuclear cells
Source: PLoS One. 2018 Oct 2;13(10):e0204827. doi: 10.1371/journal.pone.0204827 (PMC6168128; doi:10.1371/journal.pone.0204827)
Supplement: S3 Table — In three experiments on 4 cows, PBMC were immediately labelled with CFSE and either stimulated with LPS or kept as untreated control. After 3 to 6 days in culture, lymphocytes were stained with mAb to bovine CD3, CD4 and sIgM, followed by anti-mouse IgG1 PE or anti-mouse IgG2 PE. (DOCX) [file pone.0204827.s003.docx]

**S Table 3**

**Staining of bovine PBMC after CFSE labeling and LPS stimulation**

| **Gating on CFSE+, proliferated lymphocytes** | | | |
| --- | --- | --- | --- |
|  | **% CD3+** | **% CD4+** | **% sIgM+** |
| **Cow 1, LPS** | 28.3 | 6.7* | 19,8 |
| **Cow 1, control** | 20.2 | 5.7* | 16.2 |
| **Cow 2, LPS** | 53.9 | 0* | 39.0 |
| **Cow 2, control** | 61.8 | 0* | 24.5 |
| **Cow 3, LPS** | 22.2 | 1* | 58.3 |
| **Cow 3, control** | 45.2 | 1.8* | 50.3 |
| **Cow4, LPS** | 43.3 | 2* | 33.8 |
| **Cow 4, control** | 34.8 | 3.3* | 44.2 |

In three experiments on 4 cows, PBMC were immediately labelled with CFSE and either stimulated with LPS or kept as untreated control. After 3 to 6 days in culture, lymphocytes were stained with mAb to bovine CD3, CD4 and sIgM, followed by anti-mouse IgG1 PE or anti-mouse IgG2 PE. Proliferated cells (halved green fluorescence) defined a gate for the evaluation of CD3, CD4 and IgM surface expression against the background (cells reacted with either anti-mouse IgG1 PE or anti-mouse IgG2 PE, only).

*Down-regulation of surface CD4 after in vitro culture of PBMC from cows 1 (three days), 2 (six days), 3 and 4 (three days), respectively.
